# Utilization of carbon-coated ZrO_2_/Mn-Mg-Zn ferrites nanostructures for the adsorption of Cs (I) and Sr (II) from the binary system: kinetic and equilibrium studies

**DOI:** 10.1186/s13065-023-01069-z

**Published:** 2023-11-04

**Authors:** M. I. A. Abdel Maksoud, G. A. Murad, H. S. Hassan

**Affiliations:** 1 Radiation Physics Department, National Center for Radiation Research and Technology (NCRRT), Egyptian Atomic Energy Authority (EAEA), Cairo, Egypt; 2 Hot Laboratories and Waste Management Center, Egyptian Atomic Energy Authority (EAEA), Inshas, 13759 Egypt

**Keywords:** Carbon, Spinel ferrite, ZrO_2_, Adsorption, Kinetic studies, Isotherm studies

## Abstract

Carbon-coated ZrO_2_/Mn-Mg-Zn ferrites nanostructures (CZ-FN) have been prepared as a new inorganic sorbent to remove Cs (I) and Sr (II) from a waste stream. Adsorption of Cs (I) and Sr (II) has been implemented considering different noteworthy parameters, for example, shaking time and the optimum time achieved high adsorption capacity of both ions [103 and 41 mg/g for Sr (II) and Cs (I)] was found 30 min. Also, the impact of pH values was studied; the best pH value for the adsorption process is pH 6. The adsorption saturation capacity of CZ-FN is 420.22 and 250.45 mg/g for strontium and cesium, respectively. The solubility percentage of CZ-FN was calculated utilizing diverse molarities from HNO_3_, HCl, and NaOH as eluents, the obtained data reveals an increase in the solubility percentage with more increase in the molarity of the eluents. The elevation in the solubility percentage follows the following order; HNO_3_ < HCl < NaOH. The kinetic studies were applied using the nanolinear form of different kinetic models; it was found that the adsorption process obeys the nonlinear pseudo-second-order. According to equilibrium studies, the Langmuir model has been more accurate than the Freundlich model for adsorption in the case of binary systems. The values of Di for the strontium and cesium are 10^−10^ m^2^/s, which displays the chemisorption nature of this process. The greatest values of the desorption process for the strontium and cesium are 96.87% and 94.43 by 0.3 M of HNO_3_. This indicated that the carbon-coated ZrO_2_/Mn-Mg-Zn ferrites could be regenerated and recycled to remove strontium and cesium ions from waste streams.

## Introduction

Water treatment processes eliminate impurities and unacceptable materials or significantly diminish their concentration to render the water suitable for its intended purpose. This treatment is vital to human health and enables the utilization of both potable water and irrigation [1, 2]. Nuclear power introduces an appropriate energy; however, it is accompanied by several concerns, such as nuclear waste disposal and serious consequences due to unanticipated disasters [3]. Nuclear contamination is responsible for health and safety issues [4]. These radioactive wastes have been carcinogen materials that seriously impact humans [5, 6]. Thus, disposal of these wastes has become a substantial challenge that needs to be solved relatively from the waste stream in case of appreciable attention [7, 8].

Nuclear power activities create many radionuclides such as radioactive strontium-90 (^90^Sr) as a fission material [9]. Even though it has a short half-life (64 h) The main daughter nuclide in ^90^Sr decay is ^90^Y. These radionuclides release significant beta energy with a maximum value of 2.28 MeV. Zirconium-90, a stable isotope, results from ^90^Y decay [10, 11]. Many strategies for removing radionuclides from the waste stream have been used, including ion exchange [11], chemical precipitation, membrane filtering [10], solvent extraction [12, 13], and adsorption [4]. Each technique has benefits and drawbacks depending on process efficiency, ease of use, cost, and flexibility. Unfortunately, most of these technologies have drawbacks, such as high costs or insufficient removal the hazardous waste [14]. The adsorption process is the most effective option because of its great effectiveness and ease of use [15]. The adsorption of Sr (II) and Cs (I) from waste streams onto different adsorbent materials has been described by some researchers. Zhimin et al. studied the efficient removal of Cs (I) from high-level liquid wastewaters (HLLW) using calix[3] biscrown-6 functionalized millimeter-sized hierarchically porous carbon spheres. The obtained maximum adsorption capacity of Cs (I) was 22.72 mg/g [16]. Li et al. [15] investigated the efficiency of kaolin, activated carbon, bentonite, montmorillonite, attapulgite, and zeolite for the Sr (II) adsorption. The results showed that zeolite was the most highly effective sorbent, with a maximum adsorption capacity of 4.07 mg/g. The adsorption behavior of spent bleaching clay impregnated with 3-amino-5-hydroxypyrazole was studied for Y (III) removal. The results illustrated that the impregnated clay could successfully remove Y (III) from the waste stream at pH = 6.0 [17]. Umar et al. [18] prepared δ-MnO_2_ with a high surface area for rapid adsorption of Sr (II) and Cs (I). The equilibrium adsorption capacities are 58.63 and 91.42 mg/g for Sr (II) and Cs (I), respectively. Nevertheless, novel adsorbents with a high adsorption capacity and cost-effectiveness are still needed. The emergence of nanotechnology greatly impacted finding a solution to this problem by manufacturing different types of nanomaterials and using them to remove hazardous waste from the water stream. In the last decade, the discovery of nanomagnetic materials has led to wide uses in water treatment [19]. The nanomagnetic materials were examined widely in water treatment. After the sorption process, the magnetism of ferrites and their reactive surfaces allow for rapid magnetic separation. As a result, these particles may be used to remove different metal ions from aqueous solutions efficiently. Serunting et al. [20] modified magnetite nanoparticles with sodium alginate to study the efficacy of Ce (III) removal in aqueous solution. Also, Hassan et al. [21] investigated the removal efficiency of ^134^Cs and ^152+154^Eu radionuclides from nitric acid solution. The obtained data indicated that the selectivity of ^152+154^Eu radionuclides is higher than ^134^Cs at an acidic medium, and the adsorption capacities of both radionuclides are 94.75 and 18.55 mg/g, respectively. The chitosan and 2-hydroxy-5 formylbenzoic acid were supported by magnetic nanocomposite (NiFe^2^O^4^) to produce polymeric matrix (PNC) and magnetic polymer nanocomposite to enhance its stability and affinity toward radioactive Cs (I) and Sr (II) The surface area of NiFe_2_O_4_, and NiFe_2_O_4_@PNC was found to be 78.12, and 254 m2/g for NiFe^2^O^4^, and NiFe_2_O_4_@ PNC respectively. The NiFe_2_O_4_@PNC was used for the adsorption of radioactive Cs (I) and Sr (II), and the adsorption capacity was found to be 232.12 mg/g and 212.5 mg/g, respectively [22]. Abdel Maksoud et al. [23] synthesized a zinc ferrite-humic acid nanocomposite. The optimum conditions for adsorption of Ba (II) and Cs (I) onto zinc ferrite-humic acid composite were achieved as pH 5, and the adsorption capacities of the magnetic nanocomposite were 62.33 mg/g for Ba (II) and 42.55 mg/g for Cs (I).

In an earlier investigation [7], using batch procedures, we recorded the synthesis of carbon-coated zirconia/spinel ferrite nanostructures (C-coated ZrO_2_/Mn_0.5_Mg_0.25_Zn_0.25_FeO_4_) as an adsorbent to remove Co and Eu radionuclides. The XRD patterns confirmed that the C-coated ZrO_2_/Mn_0.5_Mg_0.25_Zn_0.25_FeO_4_ composite was successfully prepared. Transmission electron microscopy (TEM) also validated the composite's heterogeneity structure.

Therefore, in the present study, a magnetic nanostructured adsorbent was fabricated. The fabricated nanocomposite was well characterized and used for the adsorption of Cs (I) and Sr (II) from an aqueous solution. The interaction between the adsorbent material and both ions was determined using adsorption kinetics and adsorption isotherms models. The regeneration results revealed that the sample showed promising adsorption capacity after four cycles with Cs (I) and Sr (II). The results demonstrate that the fabricated carbon-coated ZrO_2_/Mn-Mg-Zn ferrites nanostructures can be used as an advanced and efficient adsorbent for adsorbing toxic elements from contaminated water.

## Experimental

### Synthesis of carbon-coated ZrO_2_/ Mn-Mg-Zn ferrites (CZ-FN)

The Fe, Zn, Mg, and Mn source materials were obtained from Sigma Aldrich. These materials include Fe (NO_3_)_3_·9H_2_O (98.0%), ZnSO_4_·7H_2_O (98%), Mg (NO_3_)_2_·6H_2_O, and Mn (NO_3_)_2_·4H_2_O (99%). They were used without any additional purification. Moreover, citric acid (C_6_H_8_O_7_, 99.57%) was employed as a fuel source. Our prior study described the C-coated ZrO_2_/Mn-Mg-Zn ferrites (CZ-FN) preparation method in detail [7]. A scanning electron microscope [SEM, (JEOL JSM-5600 LV, Japan)] was also used to create surface images of CZ-FN nanocomposite at variable vacuum without any coating at 12 kV accelerating voltage with a back-scatter detector that offered a clear insight into the morphology of the CZ-FN nanocomposite surface. The elemental composition and mapping images were obtained using the energy-dispersive X-ray analysis spectra (EDX, JEOL JSM-5600 LV, Japan). The functional groups in the CZ-FN nanocomposite were identified using Fourier transforms infrared (FT-IR) spectroscopy (NICOLET iS10 model equipment).

### Solubility studies

The solubility was studied using 0.5 g (m_1_) of the CZ-FN absorbent contact with 10 mL of different eluents such as HNO_3_, HCl, and NaOH for 24 h/ 25 °C; the two mixed phases were separated. The mass (m_2_) of dried CZ-FN has been measured. The percentage of solubility can be determined by applying the formula as follows:

1 $$S=\frac{{m}_{1}-{m}_{2}}{{m}_{1}}\times 100$$

### Adsorption analysis

Different batch studies to Cs (I) and Sr (II) adsorption from waste stream have been performed in a 25 mL glass container, including about 10 mL from ions solution contact with 0.01 g of the CZ-FN absorbent at room temperature. Specific factors, such as different pH values (1–9), the effect of different concentrations (50–500 ppm), and the impact of contact time, were discussed. The values of pH have been modified using HCl or NaOH. Different concentration of Cs (I) and Sr (II) remaining in the solution has been elucidated by atomic absorption spectrophotometer (Buck Scientific, VGP 210). Removal efficiency and adsorption capacity, as well as distribution coefficient onto CZ-FN, qt, have been computed from Eqs. 2–4:

2 $$\mathrm{Removal \,efficiency} \%=\frac{{C}_{o}-{C}_{t}}{{C}_{o}}\times 100$$

3 $${\mathrm{k}}_{\mathrm{d}} \left(\mathrm{mL}/\mathrm{g}\right)=\frac{ ({C}_{0}-{C}_{t})V}{{C}_{t}\times m}$$

4 $${\mathrm{q}}_{\mathrm{t}}=\frac{{C}_{o}-{C}_{t}}{{C}_{o}}\times \frac{V}{m}$$

where the symbols C_o_ and C_t_ clarify the concentration of Cs (I) as well as Sr (II) at initial and any at time, t, the volume of the solution represented by symbols V, L, Kd is the distribution coefficient (mL/g) finally, the weight of CZ-FN represented using the symbol m, g.

### Saturation capacity of CZ-FN

The prepared samples’ saturation capacity toward the metal ions’ adsorption was studied. 0.01 g of sample with 10 mL of ions solution (100 mg/L) was conducted overnight to be sure that the equilibrium was reached. The solid sample was separated from the liquid phase, and the concentration of ions was determined. This process was repeated several times using new volumes of metal ions (10 mL) until the adsorbent was completely saturated with the ions. The saturation capacity is expressed in Eq.  (4):

5 $$\mathrm{Saturation \,Capacity }=\frac{\sum qt}{100}\times {C}_{o}\times \frac{V}{m}$$

### Isotherm studies

The impact of Cs (I) and Sr (II) concentration has been explored under the most favorable conditions from pH and time. The isotherm studies took place in a series of glass containers, which involved a contact of 0.01 g of CZ-FN with 10 mL of the binary system, and this mixture was shaken. The isotherm behavior of cesium and strontium was studied at values ranging between 50 and 500 ppm. At equilibrium, a certain solution volume is pulled (1 mL), and the concentration remaining in the liquid phase is estimated. The adsorption capacity (q_e_) onto the surface of the CZ-FN at equilibrium is calculated from the following equation:

6 $${q}_{e}=\frac{{C}_{o}-{C}_{e}}{m}\times V$$

where Ce refers to equilibrium concentrations, the two isotherm models, Freundlich and Langmuir, have been applied to discuss the adsorption behavior of Sr (II) and Cs (I) at equilibrium.

## Results and discussion

### Characterization of CZ-FN

**Fig. 1:**
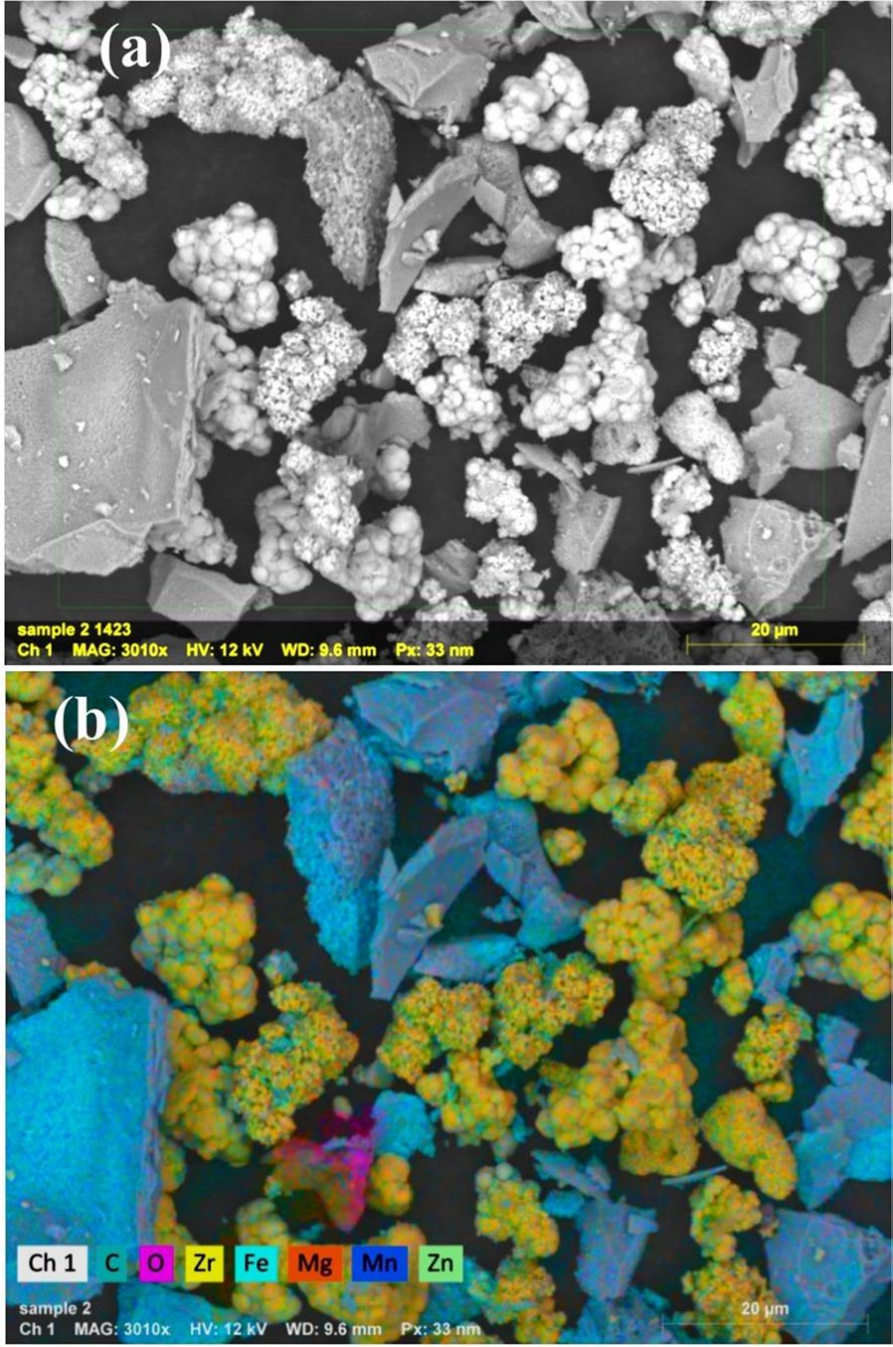
**a** SEM image of CZ-FN nanostructures, and **b** EDX mapping image of CZ-FN nanostructures

The morphology of the CZ-FN absorbent was shown using scanning electron microscopy analysis. SEM images of the CZ-FN absorbent are shown in Fig. 1a. The shape showed that the composite had a heterogeneous appearance. The ZrO_2_ particles have a rhombus form; the image indicates they agglomerate. In addition, the spinel Mn-Mg-Zn ferrite shows a porous layer nature. As shown in Fig. 1b, a mapping image was also used to determine the elemental composition of the C-coated ZrO_2_/Mn-Mg-Zn ferrite. The carbon (C), zirconium (Zr), oxygen (O), manganese (Mn), magnesium (Mg), zinc (Zn), and iron (Fe), which can be seen in the image, exist in the C-coated ZrO_2_/Mn-Mg-Zn ferrite.

**Fig. 2:**
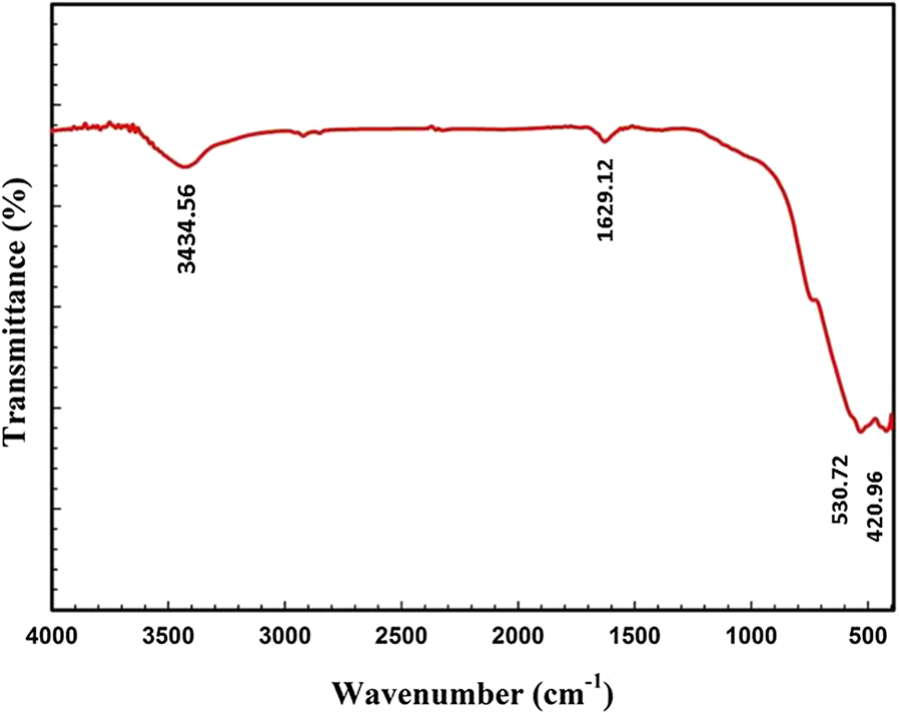
FTIR spectra of the CZ-FN nanostructures

The FTIR spectra of the CZ-FN nanostructures are depicted in Fig. 2. Two distinct vibrational bands are associated with the stretching vibration of tetrahedral groups (A-site) and octahedral groups (B-site) in spinel ferrites. The band at υ_1_ = 530 cm^−1^ corresponds to the A-site stretching, whereas the band at υ_2_ = 420 cm^−1^ corresponds to the B-site stretching in spinel MnMgZnFe_2_O_4_ NPs. Also, at around 3428 cm^−1^, O–H stretching appears. Aromatic C=C stretching was determined to be responsible for the 1635 cm^−1^ absorption band [23], indicating that the cubic spinel phase was effectively manufactured for the CZ-FN nanostructures.

### Solubility studies

**Fig. 3:**
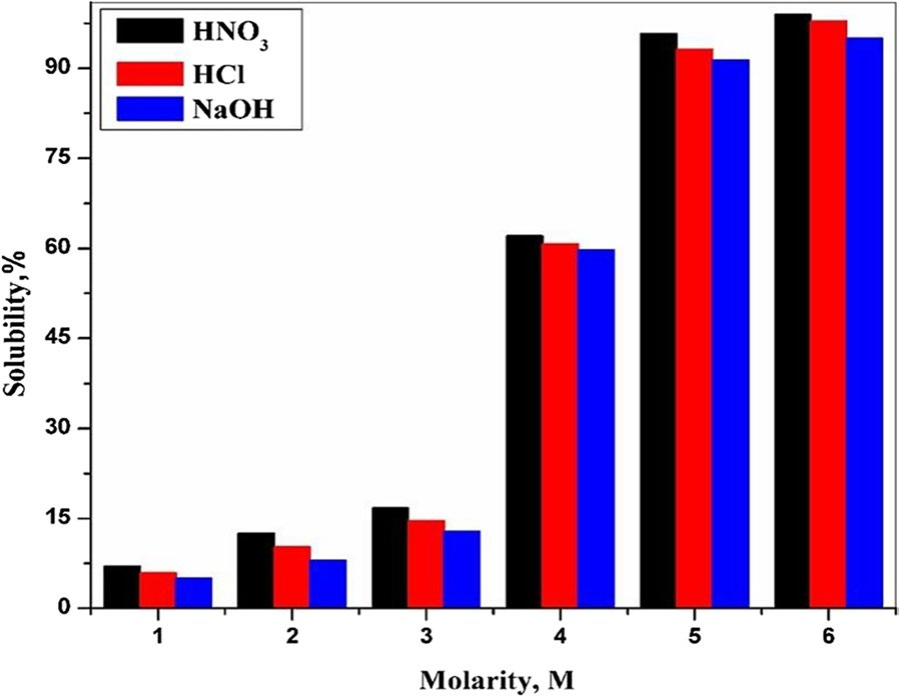
Solubility of CZ-FN

The solubility percentage of CZ-FN was calculated utilizing diverse molarities from HNO_3_, HCl, and NaOH as eluents, and the results were exhibited in Fig. 3; the obtained data reveals an increase in the solubility percentage with more increase in the molarity of the eluents. The elevation in the solubility percentage follows this order: HNO_3_ < HCl < NaOH. As shown in Fig. 3, at the lower molarity of eluents, 1 M, the solubility percentage is lower than 10%, continuously increasing eluents’ molarity until it reaches 90%.

### Saturation capacity of CZ-FN

The saturation capacity for the G has been studied and calculated through the following equation:

The obtained saturation capacities for Cs (I) have been 100.45 mg/g onto CZ-FN, whereas 220.22 mg/g for CZ-FN for Sr (II).

### Impact of CZ-FN mass

**Fig. 4:**
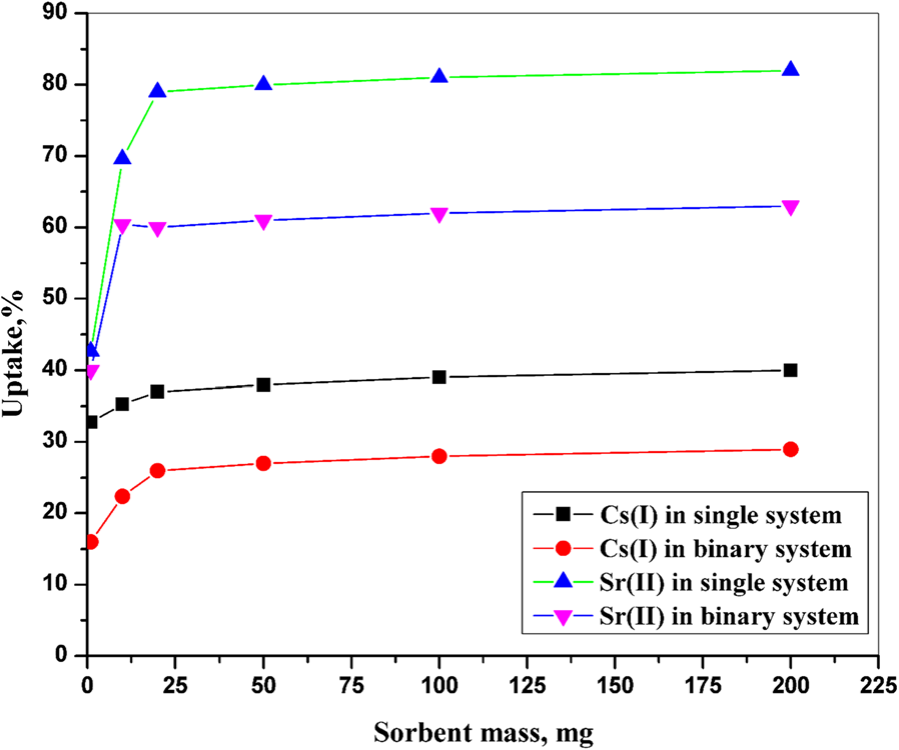
Impact of sorbent mass on Cs (I) and Sr (II) adsorption

The effect of the adsorption capacity onto the surface of the sample was studied on the adsorption process for the single and binary systems at an initial concentration of 100 ppm**, **Fig. 4. This study was carried out in a range from 1 to 200 mg of the prepared material and the volume used from the ions solution equal 10 mL. As the equilibrium case was reached, the centrifuge of the ions solutions was carried out; after that, the concentration of ions in the solution was determined. As shown in the figure, the greater the adsorption capacity, the greater the removal efficiency of ions until it becomes almost constant. The most appropriate adsorption capacity of ions onto CZ-FN has been gained at the mass of CZ-FN equal to 10 mg. The growth capacity of the adsorption process with increasing amounts of CZ-FN is predictable because as the quantity of CZ-FN rises, the active site numbers on the surface of CZ-FN increase [24]. There is an inconsistency between the removal efficiency of each ion and the amount of sorbent, and this clarifies that the functional group on the surface of CZ-FN has a marked preference for Sr (II) over Cs (I); this can be referring to increasing the hydration radius of Cs (I) comparing with Sr (II) [23].

### Impact of pH

**Fig. 5:**
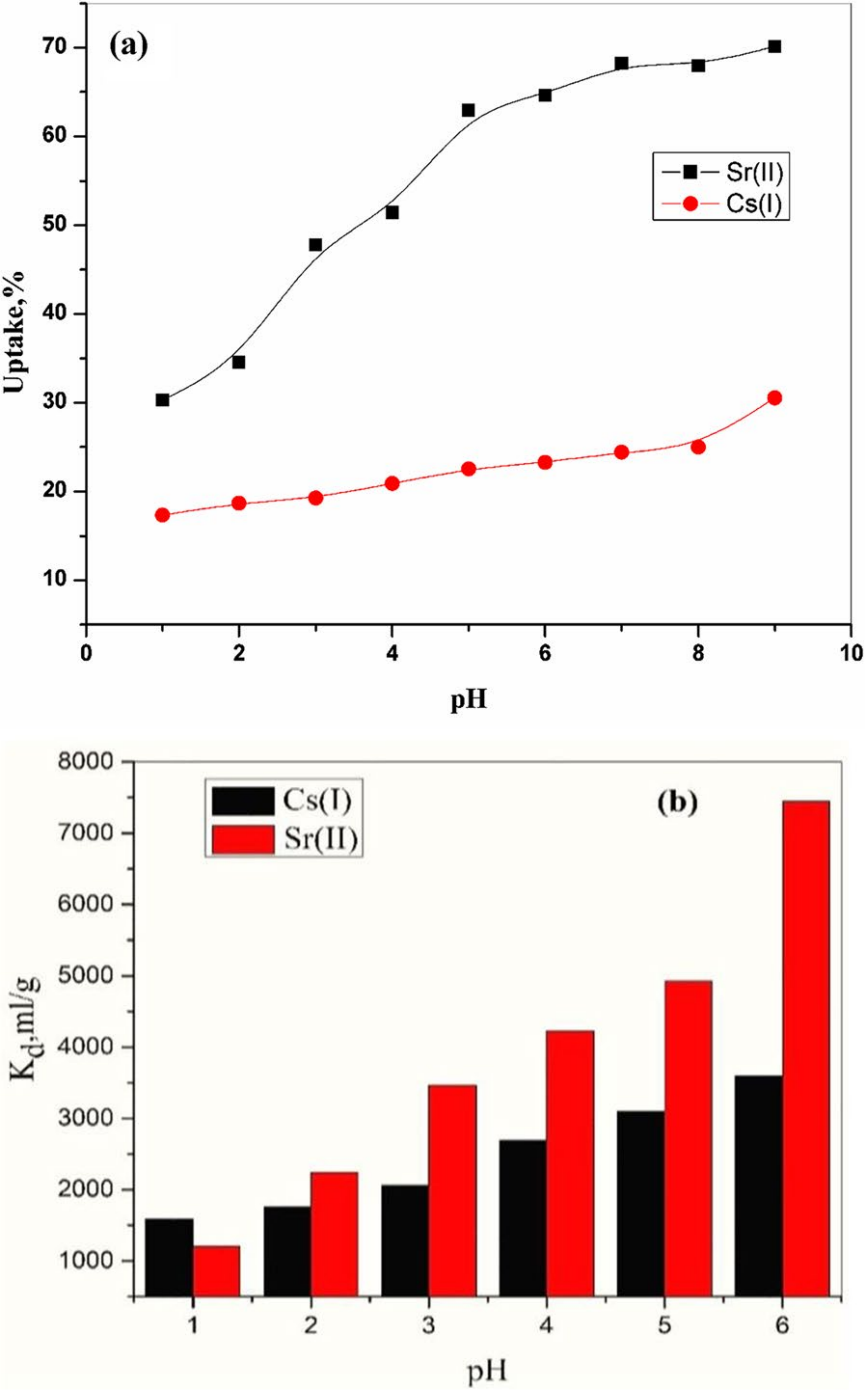
**a** Influence of pH on the sorption of Cs (I) and Sr (II) onto CZ-FN **b** the distribution coefficient of CZ-FN adsorbent in different pH

Figure 5 explains the relationship between pH values and the percentage of removal of the Cs (I) and Sr (II). This relationship increases by elevation in the pH values and chemical stability of CZ-FN powder. The lower values of the removal of Cs (I) and Sr (II) at depressed values of the pH can be elucidated; at depressed pH values, the concentration of hydrogen proton (H^+^) in the solution is elevated; thus, this proton may be competing with the Cs (I) and Sr (II) to occupy the active sites exists on the surface of CZ-FN. As elevated pH values, the competition between H^+^ proton and studied ions becomes weak; therefore, the elimination of Cs (I) and Sr (II) becomes more excellent. The static batch equilibration technique computed distribution coefficients Cs (I) and Sr (II) in aqueous solutions on CZ-FN. A series of 0.01 g of sorbent was shaken with 10 mL of 100 mg/L metal ion solution at different pH values (1.0–6.0) and room temperature overnight. After equilibration, the mixtures were centrifuged at 5000 rpm, and then 1.0 mL of the sorbate was withdrawn and measured by atomic absorption spectrophotometer (Buck Scientific, VGP 210). All measurements were carried out in duplicates. The distribution coefficients of both ions were computed by Eq.  (3). The values of K_d_ increase with the increase in pH values. At lower pH values (pH ≤ 3), Kd of both ions was inhibited. At higher pH (pH N 3), the Kd values continuously increase with the increase in pH. The K_d_ values for Sr (II) onto CZ-FN at pH 6.0 were found to be 7446 mL/g higher than the Kd values for Cs (I) (3601 mL/g).

### Adsorption studies

**Fig. 6:**
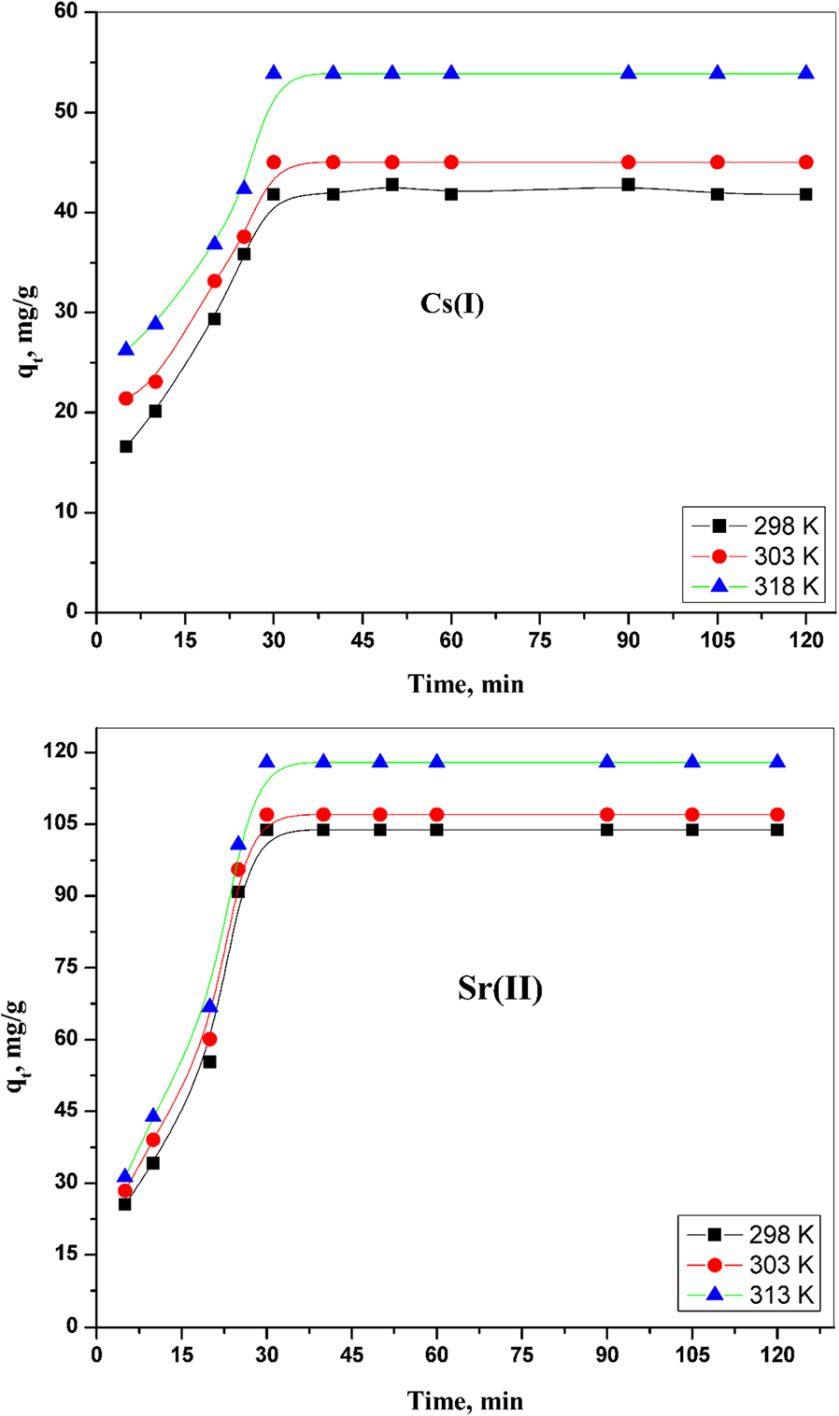
Effect of contact time on Cs (I) and Sr (II) adsorption onto CZ-FN at various temperatures

Figure 6 exhibits the plot between the amount adsorbed of Sr (II) and Cs (I) for the binary system and contact time at several temperatures. The adsorption behavior of both ions in the binary system has been rapid in the beginning process; after that, the rate of this process converts to a slow rate until an equilibrium case takes place. The adsorption of both ions increased with the contact time rising till equilibrium. The adsorption capacity from ions has been 103 and 41 mg/g for Sr (II) and Cs (I) at equilibrium, respectively. The equilibrium case can be attained after 30 min for each studied ion in this binary system.

### Kinetic studies

The study of the adsorption process is one of the most essential studies in accurately describing the adsorption rate and finding the optimal conditions for use in various fields [12]. This study used two kinetic models, the first and the second pseudo-model. The most crucial difference between these two models is the difference in the adsorption mechanism. In the first pseudo-model, each active site in the CZ-FN can adsorb only one molecule, and therefore, the adsorption mechanism is physical sorption. Nevertheless, the pseudo-second model differs from the pseudo-first model in that one molecule was adsorbed onto more than one active site, so the mechanism of the adsorption process, in this case, is chemisorption [25]. Three models were used in this work to evaluate the adsorption process of the ions used. These models are the pseudo-first and second models and the intraparticle diffusion models.

#### Pseudo-first-order model

**Fig. 7:**
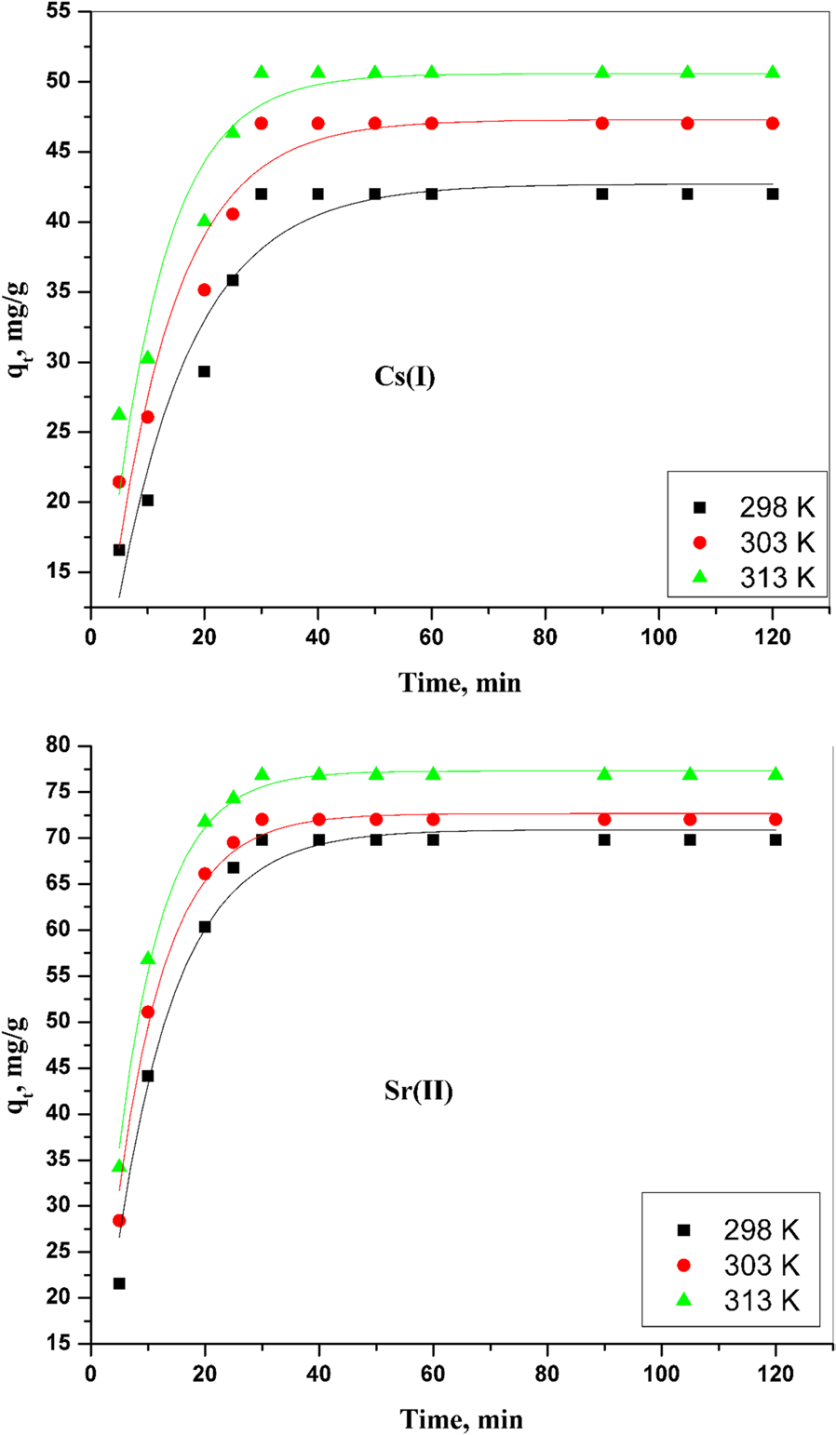
Pseudo-first order kinetic model for Cs (I) and Sr (II) sorption onto CZ-FN adsorbent

The first model imagines that the adsorption rate of Cs (I) and Sr (II) is restricted by only one mechanism on a single group from adsorbing sites; these sites are time-dependent. The adsorption rate relies on the adsorption capacity for Cs (I) and Sr (II). The non-linearized formula of this model can be expressed as shown in Eq. 7 [25]:

7 $${q}_{t}={q}_{e} \left(1-{e}^{-k1 .t}\right)$$

where qt and qe represent the amount of cesium and strontium that adsorbed onto a surface of CZ-FN at any time, t, and in the equilibrium state, whereas k_1_, min^−1^ is the rate constant of the first model. The relationship between qt and t is shown in Fig. 7. Through this relationship, the parameters of this model (k_1_ and q_e_) can be calculated, and these data are illustrated in Table 1**,** as well as the values of R^2^. As shown in Table 1, the calculated qe values are not close to their experimental value. Also, the R^2^ is less than 0.95. As shown in Table 1, the calculated qe values do not come close to their experimental value; the R^2^ is less than 0.95. Therefore, these data suggest that the adsorption of cesium and strontium onto CZ-FN does not follow this model.

**Table 1:** Calculated parameters of the studied kinetic models for adsorption of Cs (I) and Sr (II) onto CZ-FN adsorbent

Metal ions	Temp., K	First-order kinetic parameters			*q*_*e (exp)*_* (*mg/g)	Second-order kinetic parameters			Elovich parameters		
		*k*_1_ (min^−1^)	*q*_*e*_ (cal.) (mg/g)	*R*^*2*^		*k*_2_ (× 10^–3^) (g/mg.min)	*q*_*e*_(cal.) (mg/g)	*R*^*2*^	*α*		*R*^*2*^
Cs (I)	298	0.074	22.71	0.913	41.81	0.101	48.22	0.998	50.9	0.121	0.985
	303	0.087	25.28	0.913	45.03	0.129	51.52	0.999	74.8	0.132	0.985
	313	0.117	35.51	0.931	53.87	0.163	55.45	0.998	40.6	0.181	0.952
Sr (II)	298	0.094	70.91	0.945	103.08	0.131	98.20	0.999	11.3	0.059	0.977
	303	0.115	72.67	0.944	107.01	0.171	102.82	0.998	45.4	0.066	0.981
	313	0.127	77.31	0.964	117.11	0.147	112.21	0.998	37.8	0.076	0.908

#### Pseudo-second-order model

**Fig. 8:**
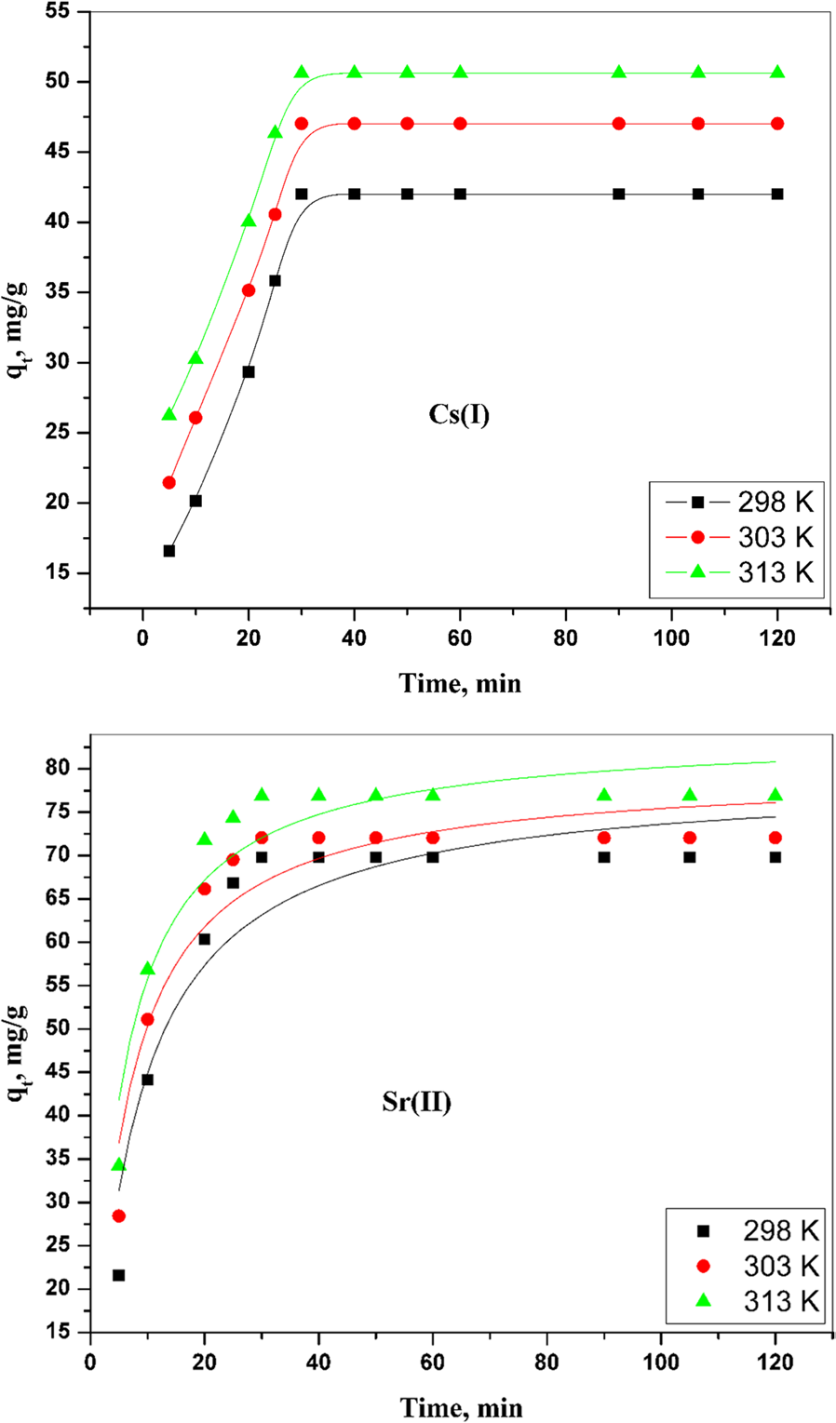
Pseudo-second order kinetic model for sorption of Cs (I) and Sr (II) onto CZ-FN adsorbent

This second model has been utilized to represent the adsorption kinetic for the system of cesium and strontium onto CZ-FN. This model has postulated a chemical rate Associated with the elimination process of the ions and chemisorption [23]. The second model has been expressed in the non-linearized formula as shown in Eq. 8 [25]:

8 $${q}_{t}=\frac{ ({k}_{2} qe2.t)}{ (1+ ({k}_{2}qe.t)}$$

where K_2_ exemplifies the constant of this model. Graphic plotting between qt and t for both Cs (I) and Sr (II) is exhibited in Fig. 8. Table 1 offers the values of the calculated parameters of this model. It is clear that The calculated amount of cesium and strontium adsorbed onto CZ-FN is close to the experimental data, and R^2^ has a higher value (0.99) than R^2^ of pseudo-first order. Thus, this model fits Cs (I) and Sr (II) adsorption processes.

#### Elovich model

**Fig. 9:**
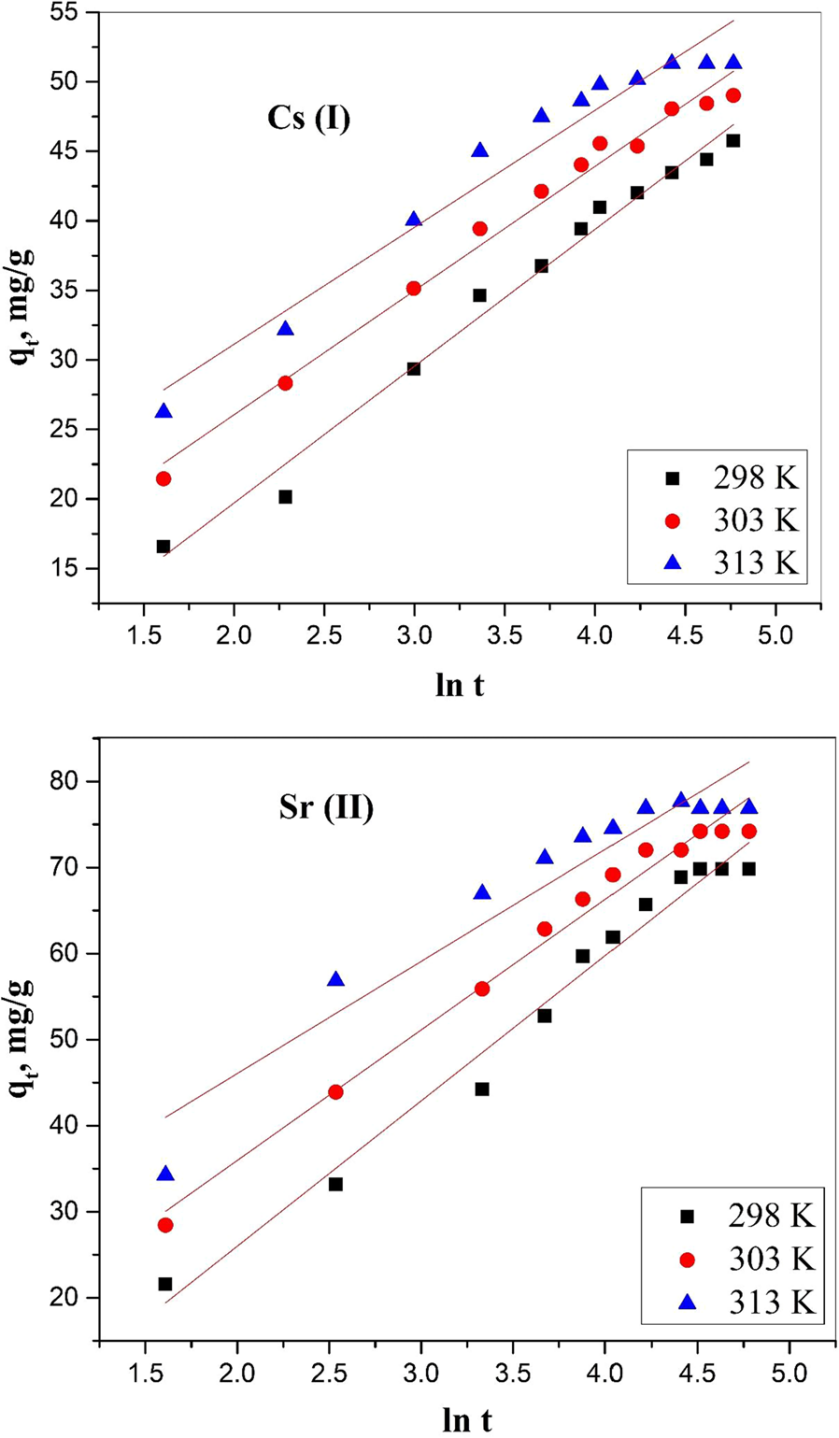
Elovich model for sorption of Cs (I) and Sr (II) onto nanocomposite material

Elovich equation is applied for chemisorption kinetics. It is often evaluated for heterogeneous surfaces [27] and devised as in Eq.  (9):

9 $${q}_{t}= \frac{1}{\beta }\mathrm{ln}\left(\alpha \beta \right)+\frac{1}{\beta }\mathrm{ln}t$$

where α and β are the Elovich constants. α (mg g^−1^ min^−1^) represents the initial sorption rate and β expresses the desorption constant (g mg^−1^) during any experiment related to the extent of surface coverage and the activation energy involved in chemisorption. The calculated Elovich parameters (α, β) from Fig. 9 and the kinetic data are illustrated in Table 1. Lamis and Gamal [11] proposed that the constant α is related to the rate of chemisorption, and constant β is related to the surface coverage. These data are consistent with the Elovich model involving Cs (I) and Sr (II) chemisorption onto the nanocomposite material.

#### Boyd model

This model concerns whether the adsorption mechanism is a film or particle diffusion. Many parameters can be known using this model by applying the following equations [27]:

10 $$F\left( t \right) = 1 - \frac{6}{{\pi ^{2} }}\sum\nolimits_{{n = 1}}^{\infty } {\frac{1}{{n^{2} }}} \exp \left( {\frac{{ - D_{i} \pi ^{2} tn^{2} }}{{r_{o}^{2} }}} \right)$$

11 $$F\left(t\right)=1-\frac{6}{{\pi }^{2}}\sum\nolimits_{{n=1}}^{\infty }\frac{1}{{n}^{2}}\mathrm{exp} (-{n}^{2}Bt)$$

12 $$B=\frac{{\pi }^{2}{D}_{i}}{{r}_{o}^{2}}$$

where F (F = q_t_/q_e_) reviews the relationship between the amount of the absorbed substance at any time, t, and at equilibrium state, also B symbolizes a constant that depends on time, while Di symbolizes the diffusion coefficient of the ions, r_o_ represents the radius of the particles; finally, n is considered an integer. The relationship between Bt and t explains whether the adsorption of Cs (I) and Sr (II) is a film or particle diffusion:

13 $${B}_{t}= -0.4977-\mathrm{ln} (1-F)$$

**Fig. 10:**
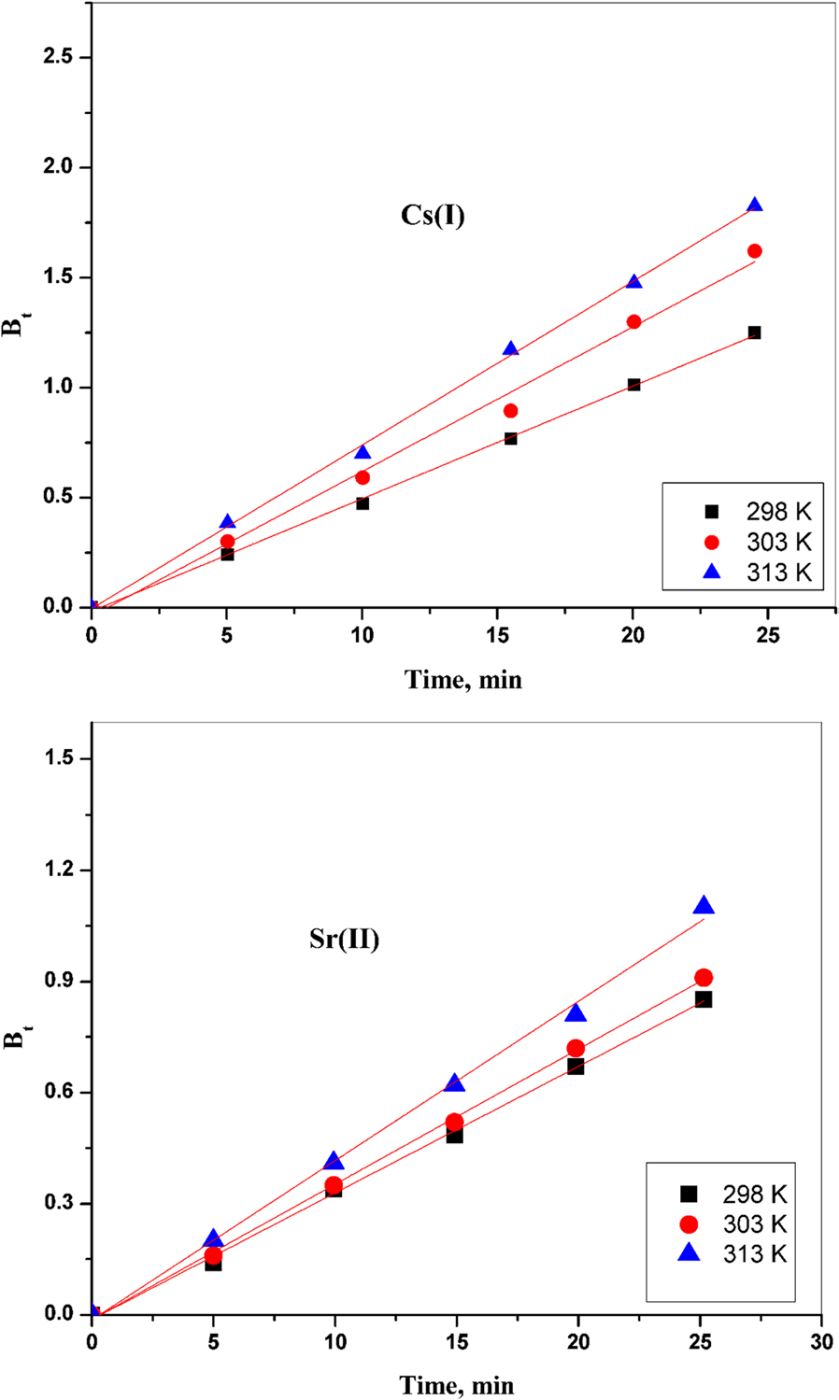
Plot of B_t_ versus Time for the adsorption of Cs (I) and Sr (II) adsorbed onto CZ-FN adsorbent

The values of F are obtained from the Reichenberg’s table [25], and the relationship between Bt and t has been used to determine if the adsorption of cesium and strontium ions takes place through film or particle diffusion. As shown in Fig. 10, plotting the relationship between B_t_ and t for Cs (I) and Sr (II) ions, a straight line relationship that passes through the point of origin. Therefore, the rate of the adsorption process is controlled by the particle diffusion mechanism. The diffusion coefficient values for both Cs (I) and Sr (II) are calculated from the slope of this straight line. Table 2 shows the obtained data.

**Table 2:** Diffusion coefficient and activation energy values for the adsorption of Cs (I) and Sr (II) onto CZ-FN adsorbent

Metal ion	D_i_ × 10^10^, m^2^/s			R^2^			D_o_ × 10^7^ m^2^/s	E_a_ kJ/mol	R^2^
	298 K	303 K	313 K	298 K	303 K	313 K			
Sr (II)	9.113	10.571	12.743	0.998	0.997	0.987	4.155	26.111	0.995
Cs (I)	11.415	12.644	14.437	0.999	0.992	0.997	4.454	13.234	0.996

The nature of the adsorption process is remarkably effectual to the Di. When diffusion coefficient values range between 10^–6^ and 10^–9^ m^2^/s, this indicates that the physical adsorption was current, while the chemisorption mechanism was prevalent, at the values of Di ranged between 10^–9^ and 10^–17^ m^2^/s. So, in this study, the chemisorption mechanism has been prevailing because the values of Di are 10^–10^ m^2^/s for each of the Sr (II) and Cs (I) [29, 30]. The straight relation for lnDi with 1/T gives a demonstration of the validation for the linear style to the Arrhenius equation:

14 $$\mathrm{ln}{D}_{i}=\mathrm{ln}\,{D}_{o }-\left(\frac{{E}_{ac}}{RT}\right)$$

The E_ac_ exemplifies the activation energies for under-studied ions, while Do represents a pre-exponential constant; these parameters can be determined by the slope and intercept of the straight plots. The gained results have been clarified in Table 2. The data acquired of the E_ac_ for strontium and cesium equal 26.11 and 13.23 kJ/mol, respectively. From these data, it is understandable that the adsorption was controlled by an intra-particle diffusion mechanism [28], which is appropriate with the acquired data above.

### Adsorption isotherm studies

The existence of more than one ion in solution shows the competition among various ions towards the adsorption active sites onto CZ-FN absorbent. Two equilibrium isotherm models have been studied to depict the adsorption of Sr (II) and Cs (I).

#### Langmuir model

The Langmuir model has been utilized to depict the binary system. The equation of this model can be written as shown in the following equation:

15 $${q}_{e}=\frac{{Q}^{0}b{C}_{e}}{1+b{C}_{e}}$$

C_e_ symbolizes the equilibrium concentration, while qe refers to the amount of ions adsorbed onto CZ-FN absorbent at equilibrium, mg/g. at the same time, b and Q^0^ represented the Langmuir model constants that indicated adsorption energy and the capacity of the adsorption process, respectively.

**Fig. 11:**
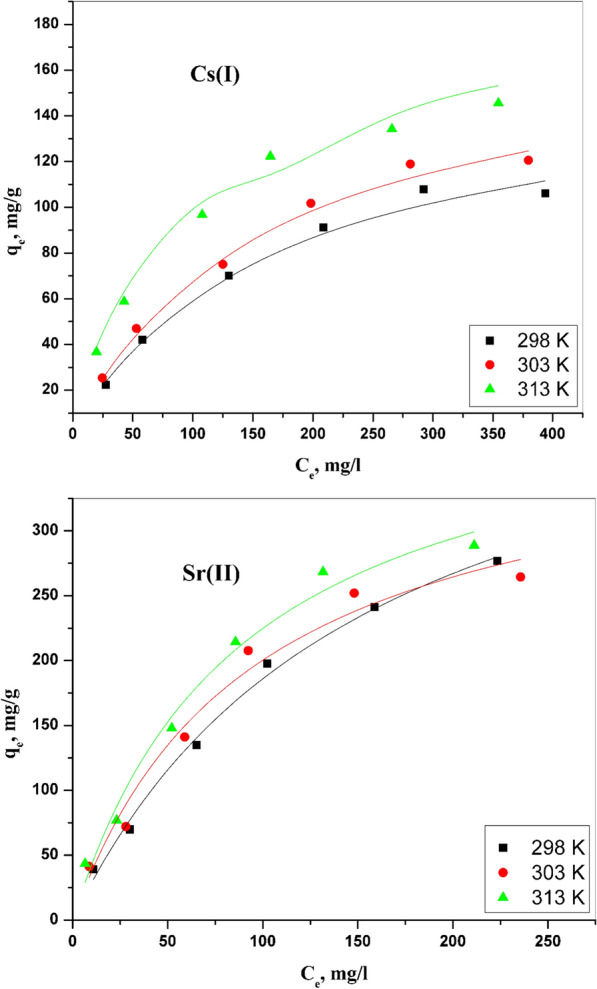
Langmuir isotherm plots for Cs (I) and Sr (II) adsorption onto CZ-FN adsorbent

The Langmuir plot has been clarified in Fig. 11, and different values for this model parameters have been evaluated and offered in Table 3. From the data in Table 3, It is intelligible that the values of Q^0^ have been increased with rising temperature. This can be explained by approachability to the active sites on CZ-FN absorbent. High values of constant b indicate the affinity of the prepared CZ-FN to sorbate. The difference between the b values for cesium and strontium indicates the affinity of the prepared material from strontium in this binary system. These obtained data are agreeable with another hypothesis suggested in different works; the dissimilarity in affinity may be due to the diversity in ionic properties for these ions as the electronegativity of the under-studied ions and the ionic radius of these ions [31].

**Table 3:** Langmuir and Freundlich parameters for the adsorption of Sr (II) and Cs (I) onto CZ-FN adsorbent

Metal ion	Temperature K	Langmuir isotherm model			Freundlich isotherm model		
		*Q*^o^ (mg/g)	*b* (L/mg)	*R*^*2*^	*K*_*f*_ (mg/g· (mg/L)^1/n^)	*1/n*	*R*^*2*^
Sr (II)	298	389.138	0.011	0.999	10.251	0.511	0.967
	303	425.754	0.012	0.999	17.384	0.521	0.975
	313	473.511	0.064	0.989	18.988	0.624	0.967
Cs (I)	298	145.395	0.001	0.989	5.675	0.447	0.959
	303	172.304	0.001	0.989	6.491	0.518	0.966
	313	194.162	0.002	0.979	12.315	0.523	0.968

#### Freundlich model

Freundlich model is conditional on the adsorbate allocation between the prepared CZ-FN absorbent surface and liquid phase at the equilibrium case. The nonlinear expression of this model can be displayed in the following equation [30]:

16 $${q}_{e}={K}_{f}{C}_{e}^\frac{1}{n}$$

**Fig. 12:**
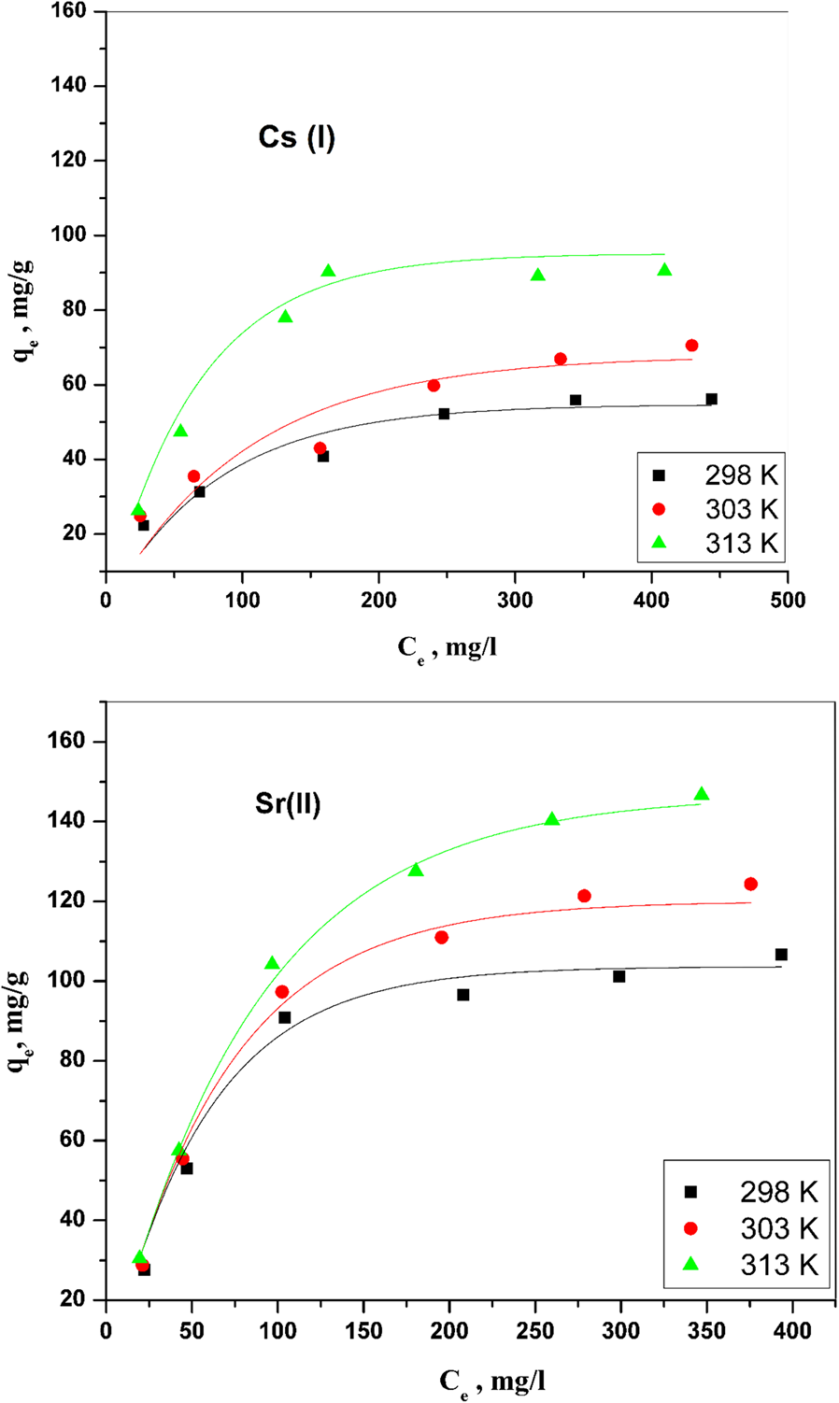
Freundlich isotherm plots for Cs (I) and Sr (II) adsorption onto CZ-FN

K_f_ is symbolized to the constant of this model that points out adsorbent capacity (mg/g· (mg/L)^1/n^), where n is a constant referring to the surface heterogeneity and can be elucidated as the adsorbent intensity. The plots of this model for the binary system are displayed in Fig. 12**,** and the different parameters of the model can be determined and clarified in Table 3. The small values of R^2^ ≥ 0.96 and the small values of K_f_ compared with the capacity and R^2^ in the Langmuir model indicate that the Langmuir model is more preferable to the Freundlich model.

### Desorption studies

Desorption studies for the sorbent are needed in many applications within the industrial field. The desorption process also clarified the efficiency of the prepared materials and, unusually, kept down the operational cost, In addition to the protection process to waste handling plants. So, in this study, the desorption experiments of under-studied ions from loaded material (CZ-FN) have been done using nitric acid and sodium hydroxide at room temperature. The impact of acid and base concentration on the desorption of cesium and strontium has been inspected employing distinct molarities from acid and base, and the range of these molarities can be located between 0.01 and 0.3 M. The desorption (D%) percentage was evaluated from the following expression:

17 $$D\%=\frac{{C}_{aq} \times 100}{{C}_{sd}}$$

**Fig. 13:**
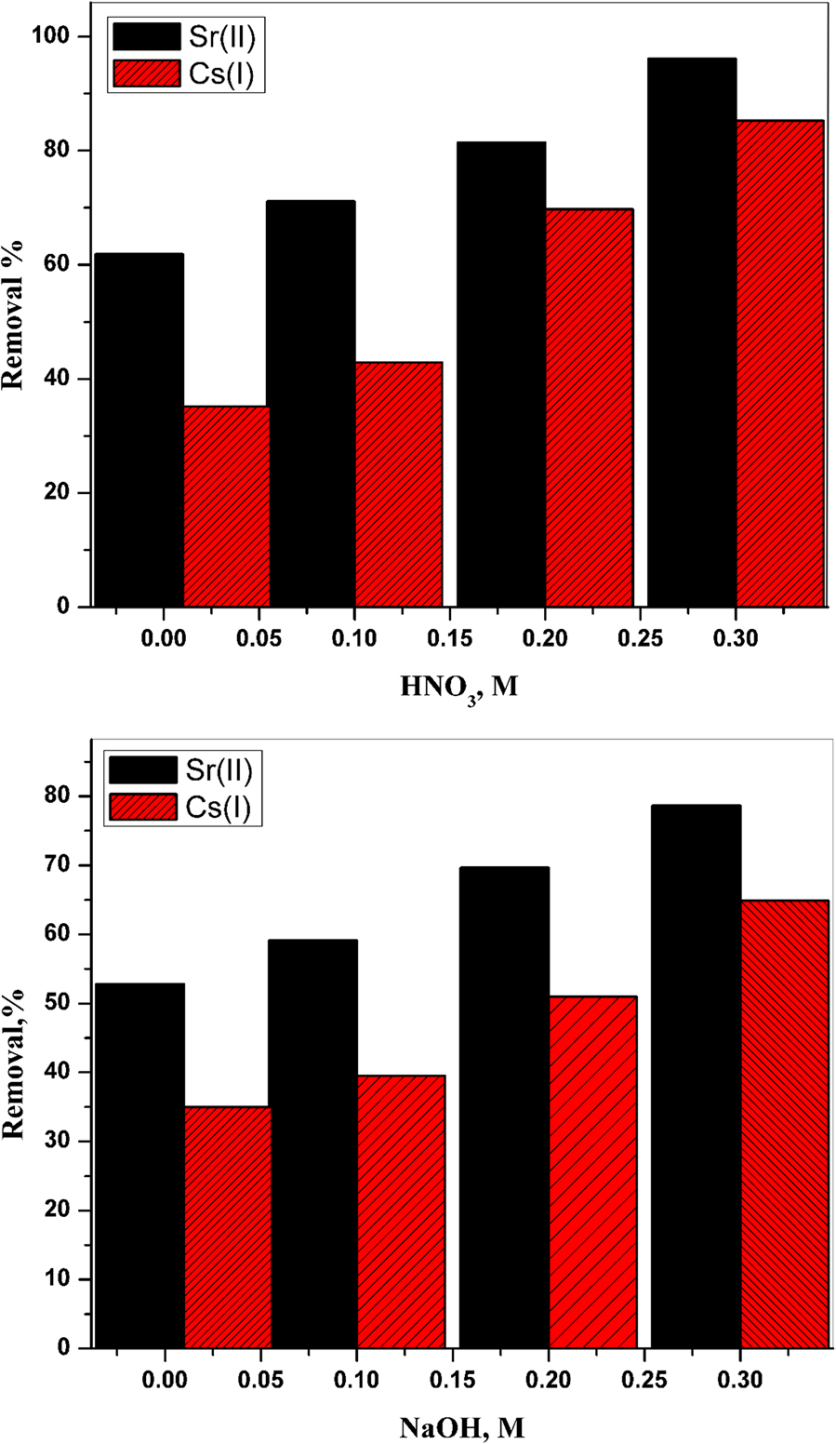
Desorption studies of Sr (II) and Cs (I) from loaded CZ-FN using HNO_3_ and NaOH

C_aq_ and C_sd_ point out concentrations of the studied ions in an aqueous solution and solid phase, respectively. The obtained data of this process illustrated in Fig. 12 elucidates that the desorption of the cesium and strontium utilizing HNO_3_ is better than that of NaOH. The extreme desorption percentage gained for both ions was 96% and 91%, utilizing a concentration from nitric acid equal to 0.3 M, Fig. 13. The regeneration of CZ-FN was done by applying the more desirable concentration of HNO_3_, 0.3M; the regeneration was carried out by four cycles, Table 4. The first cycle in adsorption has been gained by 97.87 and 83.51% for strontium and cesium, respectively, and these ratios decrease to 42.56, and 39.55% in the fourth cycle. Despite this, the percentage of desorption for both strontium and cesium after four cycles was 85.22% and 80.32%, respectively. Table 4 offers the obtained data for the adsorption–desorption percentage for all cycles. The acquired results encourage that the CZ-FN may be regenerated, and then the material can be reused for another adsorption process.

**Table 4:** Regeneration of Cs (I) and Sr (II) using 0.3 M nitric acid

Cycle no	Adsorption, %		Desorption, %	
	Sr (II)	Cs (I)	HNO_3_, 0.3 M	
			Sr (II)	Cs (I)
Cycle 1	97.99	83.76	96.87	94.43
Cycle 2	87.12	79.45	89.55	86.64
Cycle 3	65.34	65.53	88.76	84.44
Cycle 4	42.44	39.55	85.22	80.32

## Conclusion

The present study successfully fabricated a promising adsorbent based on carbon-coated ZrO_2_/Mn-Mg-Zn ferrites nanostructures, which were well characterized using several analytical techniques. The adsorption of Cs (I) and Sr (II) on CZ-FN) for the elimination of Sr (II) and Cs (I) in binary systems were studied. The adsorption kinetics and isotherms models support the nonlinear pseudosecond order and Langmuir adsorption models. The regeneration was carried out with the help of different eluents. The studied saturation capacity for the prepared CZ-FN clarified that the saturation capacity of Sr (II) equals 450.54 mg/g while Cs (I) equals 270.45 mg/g. The interaction between the metal and the adsorbent was discussed, and the overall outcomes suggest that the fabricated adsorbent can effectively remove Cs (I) and Sr (II) from contaminated water.

## Data Availability

All data generated or analysed during this study are included in this published article.
